# HDL Isolated by Immunoaffinity, Ultracentrifugation, or Precipitation is Compositionally and Functionally Distinct

**DOI:** 10.1016/j.jlr.2022.100307

**Published:** 2022-10-29

**Authors:** Michael Holzer, Senka Ljubojevic-Holzer, Douglas Ricardo Souza Junior, Julia T. Stadler, Alankrita Rani, Hubert Scharnagl, Graziella Eliza Ronsein, Gunther Marsche

**Affiliations:** 1 Division of Pharmacology, Otto-Loewi Research Centre, Medical University of Graz, Graz, Austria; 2 BioTechMed Graz, Graz, Austria; 3 Department of Cardiology, Medical University of Graz, Graz, Austria; 4 Division of Molecular Biology and Biochemistry, Gottfried Schatz Research Center, Medical University of Graz, Graz, Austria; 5 Department of Biochemistry, Institute of Chemistry, University of São Paulo, São Paulo, Brazil; 6 Clinical Institute of Medical and Chemical Laboratory Diagnostics, Medical University of Graz, Graz, Austria

**Keywords:** lipoproteins, proteomics, apolipoproteins, cholesterol/metabolism, cholesterol/efflux, density gradient ultracentrifugation, dextran-sulfate precipitation, mass spectrometry, paraoxonase 1, HDL subclass distribution, AMBP, adipocyte plasma membrane-associated protein, DIA, data independent acquisition, DS, dextran-sulfate precipitation, IA, immunoaffinity, HGR, haptoglobin-related protein, IGHG1, immunoglobulin gamma-1 chain C region, IGκC, immunoglobulin kappa chain C region, ITIH4, Inter-alpha-trypsin inhibitor heavy chain H4, PLTP, Phospholipid transfer protein, PYCOX1, Prenylcysteine oxidase 1, UC, ultracentrifugation

## Abstract

The HDL proteome has been widely recognized as an important mediator of HDL function. While a variety of HDL isolation methods exist, their impact on the HDL proteome and its associated function remain largely unknown. Here, we compared three of the most common methods for HDL isolation, namely immunoaffinity (IA), density gradient ultracentrifugation (UC), and dextran-sulfate precipitation (DS), in terms of their effects on the HDL proteome and associated functionalities. We used state-of-the-art mass spectrometry to identify 171 proteins across all three isolation methods. IA-HDL contained higher levels of paraoxonase 1, apoB, clusterin, vitronectin, and fibronectin, while UC-HDL had higher levels of apoA2, apoC3, and α-1-antytrypsin. DS-HDL was enriched with apoA4 and complement proteins, while the apoA2 content was very low. Importantly, size-exclusion chromatography analysis showed that IA-HDL isolates contained subspecies in the size range above 12 nm, which were entirely absent in UC-HDL and DS-HDL isolates. Analysis of these subspecies indicated that they primarily consisted of apoA1, IGκC, apoC1, and clusterin. Functional analysis revealed that paraoxonase 1 activity was almost completely lost in IA-HDL, despite high paraoxonase content. We observed that the elution conditions, using 3M thiocyanate, during IA resulted in an almost complete loss of paraoxonase 1 activity. Notably, the cholesterol efflux capacity of UC-HDL and DS-HDL was significantly higher compared to IA-HDL. Together, our data clearly demonstrate that the isolation procedure has a substantial impact on the composition, subclass distribution, and functionality of HDL. In summary, our data show that the isolation procedure has a significant impact on the composition, subclass distribution and functionality of HDL. Our data can be helpful in the comparison, replication and analysis of proteomic datasets of HDL.

## Graphical Abstract

**Figure:**
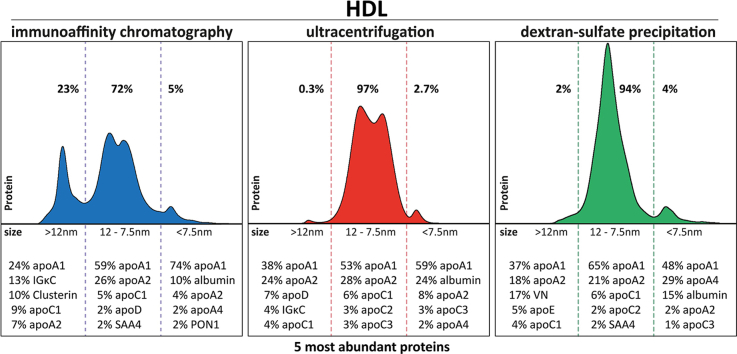

Since the discovery of HDL, a multitude of methods have been developed to isolate HDL (1). Ultracentrifugation (UC) is still the most commonly used method to isolate HDL since it was introduced by Havel *et al.* several decades ago (2). Many different versions of UC methods have been developed, and the differences are related to centrifugation time, centrifugation force, chemicals used to adjust plasma density, osmotic pressure present, and whether or not a density gradient was used. However, HDL isolation by UC is a lengthy procedure that is less suitable for clinical use. As a result, precipitation methods have been developed for the selective removal of lipoproteins from serum. These methods can also be used to isolate intact HDL particles and commonly use polyanions such as dextran sulfate (3). Precipitation of lipoproteins by dextran sulfate in the presence of divalent cations is depended on both the positive charge and negative charge of the lipoproteins protein moiety as well as the charged groups of the phospholipids present. (4) The interaction forms insoluble complexes that precipitate and can be solubilized again by the removal the reagents. In recent years, isolation of HDL by immunoaffinity (IA) chromatography using specific antibodies for apoA1 has gained importance and represents an alternative method that reflects Alaupovic's apolipoprotein-based definition of lipoprotein classes. (5, 6) Recently, Furtado *et al.* used IA chromatography to define 16 unique HDL subspecies based on the presence or absence of specific proteins besides apoA1 (7). IA chromatography has several advantages over UC, as it does not require centrifugal forces and high osmotic pressure and can isolate apoA1-containing particles from the entire size/density range of HDL. While these advantages are promising, the method also has drawbacks, such as rather low yield, high cost, and concerns about the specificity of the antibodies used. Over the past decade proteomics has expanded the list of HDL-associated proteins to over 200, while lipidomics has provided further new insights into the complexity of HDL-associated lipids (8, 9, 10, 11, 12).

A key factor in the quantification of the HDL proteome besides a precise quantification method is the isolation methodology (13). Although much effort has been invested into the development of these protocols, there is little understanding of the impact of different isolation techniques on HDL composition and function. The aim of our study was to systematically compare composition and function HDL particles isolated by common methods, such as IA, UC, and dextran-sulfate precipitation (DS). For that purpose, we used a combination of untargeted (data-dependent acquisition, DDA) and targeted (data-independent acquisition, DIA) mass spectrometry methods for precise proteomic analysis of isolated HDLs and assessed key metrics of HDL function, such as cholesterol efflux capacity, paraoxonase 1 activity, and endothelial barrier promoting activity.

## Materials and Methods

### Blood collection

Blood was sampled from 18 healthy control subjects (inclusion criteria: apparently healthy, free of chronic disease, and currently not on medication). Three independent serum pools of six participants each, matched for age and sex, were prepared from the sera of the 18 subjects (see Table 1). All subjects signed an informed consent form in agreement with the Institutional Review Board of the Medical University of Graz. All methods were carried out in accordance with the approved by the local ethics committee (Nr.: 21–523 ex 09/10) and the principles of the Declaration of Helsinki.

### HDL isolation

#### UC

Serum density was adjusted with potassium bromide (Sigma, Vienna, Austria) to 1.24 g/ml, and a two-step density gradient was generated in centrifuge tubes (16 × 76 mm, Beckman, Nr. 342,413) by layering 3 ml density-adjusted plasma (1.24 g/ml) underneath a KBr-density solution (1.063 g/ml) as described (14). Tubes were sealed and centrifuged at 65.000 rpm (415.000 *g*) for 6 h in a 90Ti fixed angle rotor (Beckman Instruments, Krefeld, Germany). After centrifugation, the HDL-containing band was collected, desalted via PD10 columns (GE Healthcare, Vienna, Austria) and either immediately used for experiments or stored with 5% sucrose at −70°C (15).

#### IA purification of HDL subspecies

HDL subspecies were isolated from human sera of healthy volunteers with modifications as described (7, 16). Serum was incubated overnight with gentle turning at 4°C with Sepharose 4B resin coupled to a polyclonal anti–apoA-I antibody (Catalog # S81-104, Fortis life science) at a ratio of 0.25 ml serum per 1 ml antibody resin. The unbound fraction was removed by washing three times with PBS. For elution, apoA1-resin was incubated for 5 min with 3 M sodium thiocyanate, and the eluate collected. Elution was repeated for a total of three times. Eluted samples were concentrated on Vivaspin Turbo 15 columns (VWR, Germany), followed by buffer exchanged to PBS on PD MiniTrap G-10 columns (Cytiva Life Science).

#### DS

We used a commercial available kit from Cell Biolabs (Nr.: STA-607). The isolation was performed according to the manufacturer’s instructions.

### Size-exclusion chromatography

NGC QUEST FPLC System (Bio-Rad, Germany) equipped with a Superdex 200 Increase 50/300 column (Nr.: 28,990,944, Cytiva Life Science) was used with DPBS containing 0.9 mM CaCl_2_ and 0.49 mM MgCl_2_, pH 7.4 as running buffer (Nr.: 14,040,133, ThermoFisher, Germany). HDL samples (0.5 mg protein) were loaded with a 0.25 ml loop and were separated with a constant flow of 0.5 ml/min. For HDL_2/3_ separations, 0.25 ml fraction was collected, and fractions pooled as indicated in Fig. 2.

### Gel electrophoresis and blotting

For native gel electrophoresis, isolated HDL (5–15 μg protein per lane) was separated by native gel electrophoresis on 4%–16% gels (BN1004BOX, ThermoFisher). Gels were run at constant voltage of 150 V for 120 min. As a high molecular weight marker (NativeMark, Nr.: LC0725, Life Technologies, Austria), containing bovine serum albumin (7.1 nm), lactate dehydrogenase (8.2 nm), B-phycoerythrin (10.5 nm, apoferritin band 1 (12.2 nm), and apoferritin band 2 (18.0 nm) was used to estimate the size of HDL. Afterwards, gels were either stained with a freshly prepared solution of Coomassie Brilliant Blue G-250 overnight (ThermoFisher) or used for blotting. Gels were transferred to polyvinylidene difluoride membranes with an iBlot 2 Dry Blotting System at 100V for 7 min at RT. Membranes were probed blocked with 5 % milk in PBS for 1 hour and incubated with the following primary antibodies diluted in 5 % milk in PBS overnight at 4°C. The list of antibodies used can be found in the supporting information (supplemental Table S1).

Membranes were washed carefully for at least three times with wash buffer and incubated with secondary HRP-conjugated antibodies (goat anti-rabbit, Nr.:111-005-045; goat anti-mouse, Nr.:115-005-146; rabbit anti-goat, Nr.:305-005-045) for 2 h at RT. Membranes were carefully washed and developed using Clarity ECL western reagents (Nr.: 170–5061, Bio-Rad, Austria). Detection was performed on a Chemidoc Touch imaging system (Bio-Rad, Austria).

### Proteomics

#### HDL digestion

HDL (5–10 μg) was solubilized in 100 mM ammonium bicarbonate in the presence of 0.2% sodium deoxycholate (Sigma-Aldrich), reduced with 5 mM dithiothreitol (Bio-Rad) and alkylated with iodoacetamide (Bio-Rad). Proteins were digested with trypsin from Promega (1:40, w:w, enzyme: HDL protein) for 4 h at 37°C. A second trypsin aliquot was added to the samples (1:50, w:w HDL protein) and incubated overnight at 37°C (17). Digestion was stopped, and sodium deoxycholate was precipitated with 0.6% trifluoroacetic acid (Sigma-Aldrich). Samples were desalted according to the StageTip protocol (18), dried under vacuum and stored at −80ºC until MS analyses. Before MS analyses, samples were resuspended in 0.1% formic acid (Fluka), with a final protein concentration of 50 ng/μl.

#### MS proteomic analyses

Digested HDL proteins (50 ng) were loaded onto a trap column (nanoViper C18, 3 μm, 75 μm × 2 cm, Thermo Scientific) and eluted onto a C18 column (nanoViper, 2 μm, 75 μm × 15 cm, Thermo Scientific). Peptides were analyzed using an Easy-nLC 1200 UHPLC system (Thermo Scientific) coupled to an Orbitrap Fusion Lumos (Thermo Scientific) equipped with a nanospray FlexNG ion source (Thermo Scientific) in a 44 min gradient and normalized collision energy of 30 for HCD fragmentation. For untargeted analysis (DDA), peptides were analyzed using MS1 resolution of 120,000 (at *m/z* 200) with AGC target set to 4 × 10^5^, m/z range of 350–1550, and maximum injection time of 50 ms. MS2 resolution was set at 30,000 (at *m/z* 200) with AGC target of 5 × 10^4^ and maximum injection time of 54 ms. For targeted analysis (DIA), peptides were quantified using Orbitrap resolution of 30,000 (at *m/z* 200) with AGC target of 5 × 10^5^, precursor m/z range of 400–900, scan range of product ions between m/z 100 and 1000, maximum injection time of 54 ms, and isolation windows of 25 *m/z* with 0.5 *m/z* margins.

#### MS data processing

MaxQuant software (version 2.0.1.0) was used to search raw shotgun MS spectra against the human proteome (Uniprot, 20,371 entries). The criteria for protein detection and quantification included at least two peptides (at least one of them unique), with methionine oxidation and protein *N*-terminal acetylation selected as variable modifications and carbamidomethylation of cysteine as fixed modification. DDA data were used to build a library for DIA analyses. DIA data were analyzed using Skyline software (version 21.1.0.146) as described (17). For DIA, we used only unique peptides (at least 4 transitions per peptide) and avoided choosing peptides susceptible to ex vivo modification (i.e., containing methionine), peptides with high interference signals and mass error higher than 10 ppm. All peaks used for quantification were manually inspected to select the best transitions and ensure correct peak detection and integration. A pooled quality control (QC) was made by combining unfractionated digested HDL samples isolated by IA, UC, and DS (n = 3 each). This QC was injected 7 times intercalating with samples to control for technical variability. Proteins and peptides that achieved coefficients of variation lower than 15% in the pooled HDL QC were considered for quantification. For each protein, quantification was performed by summing up the areas of 2–6 most intense peptides. To give an estimate of protein abundance within each HDL, the value obtained for each protein quantification was divided by the theoretical number of tryptic peptides.

### Arylesterase activity of paraoxonase-1

Ca^2+^-dependent arylesterase activity was determined with a photometric assay using phenylacetate as previously described. HDL (0.5 μg protein) was added to 200 μl buffer containing 100 mmol/L Tris, 2 mmol/L CaCl2 (pH 8.0), and 1 mmol/L phenylacetate to a 96-well quartz glass plate (Hellma, Baden, Germany). The rate of hydrolysis of phenylacetate was monitored by the increase of absorbance at 270 nm, and readings were taken every 15 s at room temperature to generate a kinetic plot. The slope from the kinetic chart was used to determine the increase in fluorescence per minute. Enzymatic activity was calculated with the Beer-Lambert Law from the molar extinction coefficient of 1310 mol^1^∗L^−1^∗cm^−1^ for phenylacetate.

### Cholesterol efflux assay

J774.2 cells were maintained in Dulbecco’s Modified Eagle’s Medium (DMEM) in the presence of 10% fetal bovine serum and 1% penicillin/streptomycin. Cells were plated on 48-well plates (300,000 cells/well), cultured for 24 h, and loaded with 0.5 μCi/ml radiolabeled [3H]-cholesterol in DMEM supplemented with 2% fetal bovine serum and 1% penicillin/streptomycin in the presence or absence of 0.3 mM 8-(4-chlorophenylthio)-cyclic adenosine monophosphate overnight to induce the expression of adenosine triphosphate-binding cassette subfamily A member 1 (ABCA1). After labeling, cells were rinsed with serum-free DMEM containing 1% penicillin/streptomycin and equilibrated with serum-free DMEM containing penicillin/streptomycin and 2 mg/ml bovine serum albumin for 2 h. Subsequently [^3^H]-cholesterol efflux was determined by incubating cells for 3 h with 50 μg protein/ml HDL. Cholesterol efflux was expressed as radioactivity in the cell culture supernatant relative to total radioactivity (in the cell culture supernatant and cells) of three independent experiments respectively, measured in duplicates. All steps were performed in the presence of 2 μg/ml of an acyl coenzyme A cholesterol acyltransferase inhibitor (Sandoz 58-035).

### Endothelial barrier promoting activity assay

96W20idf chips (Ibidi, Germany) were incubated with 10 mM L-cysteine for 10 min at RT, washed twice with PBS, followed by a coating with 1% gelatine for 30 min at 37°C. The human umbilical vein cell line (Ea.hy926) was maintained in DMEM containing 10% FBS, 1% HAT and 1% Penstrept, seeded at 30.000 per well, and grown to full confluence for two days. Cells were serum-starved prior to experiments and baseline recorded until stable, which was routinely achieved after 1–2 h. Afterward, 100 μg/ml HDL was added, and impedance monitored over a period of 20 h. For quantification, we used the impedance recorded at 4000 Hz at the 10 h time point.

### Statistical analysis

Differences between two groups (Fig. 4C, D; with and without thiocyanate treatment) were analyzed with the Student’s *t* test. Differences between three groups (IA vs. UC vs. DS) were analyzed with one-way ANOVA followed by Bonferroni’s multiple comparison test. Significance was accepted at ∗*P* < 0.05 and ∗∗*P* < 0.01. Statistical analyses were performed with GraphPad Prism, Version 6, and SPSS, Version 26. Principal component analysis was performed on Perseus (v.2.0.3.1) with imputation of missing values (less than 2% of the total).

## Results

We isolated HDL with three different methods, being IA, UC, and DS (Table 1) from three independent serum pools. While UC is based on the density difference of HDL to other serum compounds, IA directly targets the major HDL associated protein apoA1. Precipitation uses a specific interaction of polyanions with charged groups on proteins and lipids to precipitate HDL. The baseline characteristics for the serum pools used are given in the supplemental Table S2.

**Table 1:** Overview of methods used to isolate HDL

Acronym	Method	Mode of Separation	Compounds Used	Factors to be Considered	Proteins Detected by MS
IA-HDL	Immunoaffinity	apoA-I content	apoA-I antibody	- Antibody specificity	142
UC-HDL	Ultracentrifugation	density	Potassium bromide	- Osmotic pressure - Centrifugal force	112
DS-HDL	Precipitation	solubility	Dextran sulfate	- Precipitation specificity	123

DS, dextran-sulfate precipitation; IA, immunoaffinity; UC, ultracentrifugation.

To investigate the differences in the proteome of isolated HDL, we used a combination of untargeted DDA and targeted DIA mass spectrometry methods. Global protein discovery by DDA allowed us to detect a total of 171 proteins across all three isolation methods, with 142 proteins being present in IA-HDL, 112 proteins in UC-HDL, and 123 proteins in DS-HDL (Table 1 and supplemental data). We used the DDA results to construct a library to analyze DIA samples. DIA chromatograms obtained in the Skyline software were carefully analyzed, and peptides of 51 HDL proteins that were consistently and confidently detected in a pool of all samples with low coefficient of variation (<15%) were selected for label-free relative quantitative analysis. Raw data and calculations of the mass spectrometry data are provided in the Supplementary Data section.

**Fig. 1:**
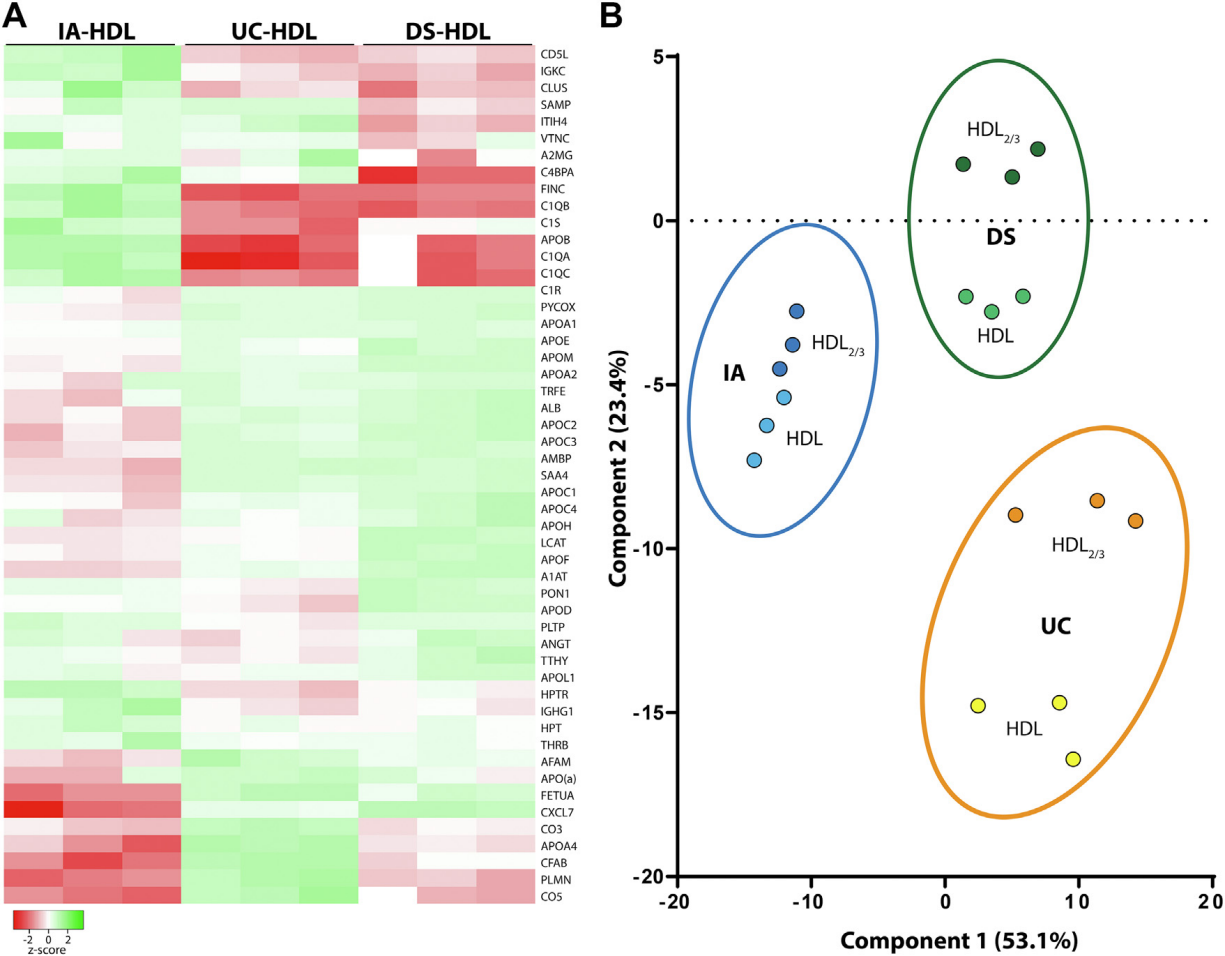
HDL isolated by different methods is compositional distinct. A: Heatmap of proteomics DIA data from HDL isolation by immunoaffinity (IA-HDL), ultracentrifugation (UC-HDL), or dextran-sulfate precipitation (DS-HDL) from three independent samples. B: Principal component analysis of HDL and HDL_2/3_. For each protein, the integrated area of the DIA analysis obtained in Skyline software was used to build the heat map and the PCA. Proteins with CV <15% in the pooled HDL quality control (n = 51) were included in PCA analysis. DIA, data-independent acquisition.

The results from the targeted DIA proteomics clearly showed that the composition of HDL from the different isolation methods is distinct (Fig. 1A). A principal component analysis of the results showed a distinct cluster for each individual isolation method indicating that the methods are indeed different (Fig. 1B). The principal component analysis clusters further suggested that the tested methods are reproducible, which was also confirmed by gel electrophoresis analysis of individual isolations from the same serum pool (supplemental Fig. S1). The results indicate that the major HDL-associated protein, apoA-I, accounts for 53.4%–59.8% of the total isolated protein and was lowest in UC-HDL (Table 2). Further changes in UC-HDL where higher levels of apoA2, apoC3, and α-1-antytrypsin, while IA-HDL had higher levels of paraoxonase 1, PLTP, apoB, clusterin, vitronectin, fibronectin as well as a series of complement proteins (Table 2). DS-HDL was markedly enriched in apoA4 and complement proteins, while the content of apoA2, apoD, and paraoxonase 1 was very low compared to the other methods (Table 2).

**Table 2:** Mass spectrometry analysis of HDL

Protein Name	Immunoaffinity	Ultracentrifugation	Precipitation
	% of Total Protein (Mean ± Stdev)	% of Total Protein (Mean ± Stdev)	% of Total Protein (Mean ± Stdev)
Apolipoprotein A1	56,8413 ± 24,209	53,4370 ± 18,337	59,7746 ± 14,581^c^
Apolipoprotein A2	21,9839 ± 14,240	25,2083 ± 06,841	18,0152 ± 08,703^a^^,^^b^^,^^c^
Apolipoprotein C1	52,658 ± 07,134	68,114 ± 08,130	79,474 ± 07,789^b^
Apolipoprotein C3	24,509 ± 06,718	43,525 ± 07,868	33,411 ± 04,694^a^
Apolipoprotein C2	25,120 ± 05,522	31,688 ± 07,108	31,557 ± 07,099
Apolipoprotein D	21,532 ± 02,495	22,346 ± 03,651	04,058 ± 00,523^b^^,^^c^
Serum amyloid A4	13,113 ± 01,883	18,542 ± 01,000	23,306 ± 02,227^a^^,^^b^
Albumin	07,869 ± 04,381	09,337 ± 02,056	07,463 ± 00,513
Paraoxonase 1	09,036 ± 00,352	06,791 ± 01,522	01,989 ± 00,144^b^^,^^c^
Apolipoprotein M	02,774 ± 00,236	02,914 ± 00,514	01,807 ± 00,236^b^^,^^c^
Apolipoprotein E	04,830 ± 00,496	02,817 ± 00,476	03,636 ± 00,735^a^
Platelet basic protein	00,148 ± 00,097	01,489 ± 00,388	00,546 ± 00,030^a^^,^^c^
Apolipoprotein A4	04,266 ± 02,291	01,245 ± 00,098	26,678 ± 02,132^b^^,^^c^
Apolipoprotein F	00,533 ± 00,078	00,797 ± 00,066	00,280 ± 00,030^a^^,^^c^
α-1-antitrypsin	00,340 ± 00,072	00,687 ± 00,201	00,302 ± 00,089^a^^,^^b^^,^^c^
Transthyretin	00,641 ± 00,063	00,547 ± 00,283	00,147 ± 00,058^b^
IGκC	17,060 ± 04,752	00,487 ± 00,011	00,735 ± 00,063
Apolipoprotein C4	00,290 ± 00,070	00,379 ± 00,110	00,186 ± 00,046
Fetuin A	00,140 ± 00,042	00,346 ± 00,169	00,841 ± 00,230^b^^,^^c^
Apolipoprotein L1	00,390 ± 00,082	00,257 ± 00,101	00,157 ± 00,070^b^
IGHG1	02,317 ± 00,872	00,201 ± 00,052	00,261 ± 00,039^a^^,^^b^
Thrombin	00,978 ± 00,382	00,175 ± 00,028	00,287 ± 00,048^a^^,^^b^
Apolipoprotein H	00,123 ± 00,062	00,138 ± 00,052	00,071 ± 00,013^a^^,^^b^
Clusterin	09,980 ± 02,971	00,131 ± 00,018	01,032 ± 00,225^a^^,^^b^
Serotransferrin	00,228 ± 00,126	00,130 ± 00,017	00,166 ± 00,007
PLTP	00,267 ± 00,020	00,094 ± 00,006	00,053 ± 00,004^a^^,^^b^^,^^c^
LCAT	00,029 ± 00,004	00,052 ± 00,018	00,015 ± 00,004^c^
Complement C3	00,256 ± 00,076	00,052 ± 00,019	00,763 ± 00,157^b^^,^^c^
Vitronectin	02,077 ± 00,372	00,049 ± 00,023	01,818 ± 00,894^a^^,^^c^
HGR	00,427 ± 00,038	00,038 ± 00,008	00,028 ± 00,005^a^^,^^b^
PYCOX1	00,039 ± 00,010	00,033 ± 00,003	00,035 ± 00,006
Protein AMBP	00,025 ± 00,006	00,032 ± 00,009	00,033 ± 00,004
Haptoglobin	00,153 ± 00,026	00,024 ± 00,007	00,042 ± 00,022^a^^,^^b^
Angiotensinogen	00,024 ± 00,008	00,016 ± 00,010	00,005 ± 00,003
Apolipoprotein B	00,150 ± 00,059	00,014 ± 00,006	00,002 ± 00,001^a^^,^^b^
α-2-macroglobulin	00,101 ± 00,064	00,010 ± 00,013	00,032 ± 00,012
Serum amyloid P-C	00,778 ± 00,437	00,008 ± 00,008	00,053 ± 00,039^a^^,^^b^
Plasminogen	00,050 ± 00,019	00,007 ± 00,002	00,474 ± 00,150^b^^,^^c^
Complement C1qB	01,505 ± 00,365	00,005 ± 00,005	00,010 ± 00,012^a^^,^^b^
CD5 antigen-like	00,297 ± 00,033	00,004 ± 00,002	00,011 ± 00,004^a^^,^^b^
Complement C1qC	02,089 ± 00,331	00,003 ± 00,006	00,007 ± 00,011^a^^,^^b^
Complement C1s	01,719 ± 00,448	00,003 ± 00,003	00,018 ± 00,005^a^^,^^b^
Complement factor B	00,004 ± 00,002	00,003 ± 00,002	00,045 ± 00,008^a^^,^^b^
Complement C1qA	01,770 ± 00,177	00,003 ± 00,005	00,009 ± 00,013^a^^,^^b^
C4b-binding protein α	00,045 ± 00,003	00,003 ± 00,005	00,036 ± 00,038
Apolipoprotein(a)	00,005 ± 00,004	00,003 ± 00,002	00,009 ± 00,001
ITIH4	00,041 ± 00,015	00,002 ± 00,001	00,027 ± 00,002^a^^,^^c^
Afamin	00,003 ± 00,001	00,001 ± 00,000	00,007 ± 00,003^c^
Fibronectin	00,434 ± 00,151	00,001 ± 00,001	00,130 ± 00,073^a^^,^^b^
Complement C1r	00,581 ± 00,147	00,001 ± 00,001	00,008 ± 00,004^a^^,^^b^
Complement C5	00,004 ± 00,001	00,000 ± 00,000	00,045 ± 00,018^b^^,^^c^

AMBP, adipocyte plasma membrane-associated protein; DS, dextran-sulfate precipitation; IA, immunoaffinity; HGR, haptoglobin-related protein; IGHG1, immunoglobulin gamma-1 chain C region; IGκC, immunoglobulin kappa chain C region; ITIH4, Inter-alpha-trypsin inhibitor heavy chain H4; PLTP, Phospholipid transfer protein; PYCOX1, Prenylcysteine oxidase 1; UC, ultracentrifugation.

a IA-HDL versus UC-HDL.

b IA-HDL versus DS-HDL.

c UC-HDL versus DS-HDL; *P* < 0.05.

**Fig. 2:**
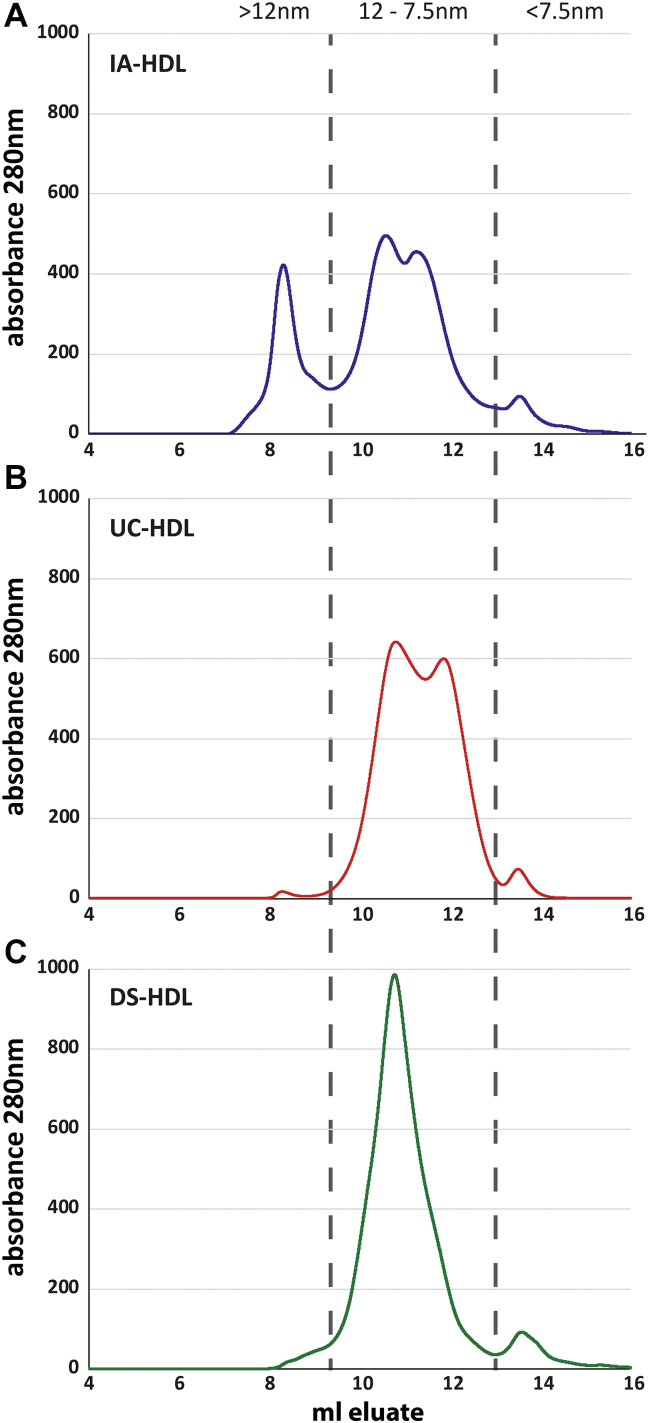
Size-exclusion chromatography of isolated HDL. Separation of IA-HDL(A), UC-HDL (B) and DS-HDL (C) was performed on an NGC Quest FPLC system equipped with a Superdex 200 column. Dashed lines represent the usual size range of the main HDL_2/3_ subclass. DS, dextran-sulfate precipitation; IA, immunoaffinity chromatography; UC, ultracentrifugation.

To investigate the size distribution of isolated HDL, we used size-exclusion chromatography (SEC). Interestingly, we found that HDL isolated by IA contained a fraction larger than 12 nm, which was almost entirely absent with the other isolation methods. The distribution between the HDL_2/3_ subclasses was similar between IA-HDL and UC-HDL, while the HDL_3_ subclass was largely missing in DS-HDL (Fig. 2). To further investigate the different subclasses, we collected three major fraction (above 12 nm, between 12 – 7.5 nm and below 7.5 nm) from the SEC for proteomics analysis (Fig. 2).

Mass spectrometry data indicated that the subclass larger than 12 nm, which was only present in IA-HDL, contained primarily apoA1 (24%), IGκC (13%), clusterin (10%), apoC1 (9%), and apoC2 (4%) together with a small amount of apoB (0.3%) (Table 3). The fraction larger than 12 nm from IA-HDL contained a large amount of various complement C1q proteins. However, recent reports have shown that this might be an artefact, at least in part, of protein isolation using sepharose as a matrix (19). The size fraction above 12 nm was barely present in UC-HDL and DS-HDL, where it was mainly composed of apoA1 and apoA2 (Tables 4 and 5). The main HDL subclasses HDL_2/3_ is located in the size range between 7.5 and 12 nm (Fig. 2).

**Table 3:** Proteome of IA-HDL separated by size-exclusion chromatography

Protein Name	>12 nm	12–7.5 nm	<7.5 nm
	% of Total Protein (Mean ± Stdev)	% of Total Protein (Mean ± Stdev)	% of Total Protein (Mean ± Stdev)
Apolipoprotein A1	24,4566 ± 55,802	59,2482 ± 13,365	74,2260 ± 28,776
Apolipoprotein A2	73,363 ± 14,840	25,6114 ± 20,961	42,585 ± 16,097
Apolipoprotein C1	91,468 ± 21,440	45,224 ± 02,184	01,224 ± 00,056
Apolipoprotein C2	40,451 ± 21,398	11,736 ± 01,844	02,017 ± 02,278
Apolipoprotein C3	24,340 ± 07,282	08,126 ± 03,080	04,682 ± 05,433
Apolipoprotein D	30,039 ± 07,605	23,492 ± 01,698	08,884 ± 01,352
Serum amyloid A4	09,561 ± 01,553	15,816 ± 00,808	00,964 ± 00,422
Paraoxonase 1	23,312 ± 03,605	06,756 ± 00,428	25,129 ± 06,302
Apolipoprotein M	02,951 ± 00,766	03,200 ± 00,317	00,310 ± 00,108
Apolipoprotein E	32,294 ± 13,026	02,116 ± 00,253	03,157 ± 01,612
Apolipoprotein F	00,416 ± 00,086	00,428 ± 00,164	00,552 ± 00,198
Albumin	05,457 ± 04,154	01,937 ± 01,030	10,2662 ± 56,717
IGκC	13,2735 ± 25,837	11,183 ± 04,238	04,061 ± 01,473
Apolipoprotein A4	06,085 ± 02,706	01,205 ± 00,833	39,967 ± 21,512
Apolipoprotein C4	00,981 ± 00,242	00,198 ± 00,042	00,001 ± 00,000
Apolipoprotein L1	00,345 ± 00,069	00,449 ± 00,074	00,074 ± 00,044
IGHG1	12,362 ± 03,601	02,274 ± 00,715	01,810 ± 00,670
Transthyretin	01,119 ± 00,171	00,338 ± 00,074	00,651 ± 00,101
Clusterin	96,794 ± 09,960	07,937 ± 01,792	04,788 ± 01,786
α-1-antitrypsin	00,346 ± 00,147	00,154 ± 00,039	02,789 ± 00,227
PLTP	01,554 ± 00,156	00,247 ± 00,029	00,029 ± 00,008
LCAT	00,059 ± 00,014	00,017 ± 00,005	00,176 ± 00,037
PYCOX1	00,142 ± 00,064	00,032 ± 00,007	00,197 ± 00,090
HGR	02,184 ± 00,242	00,445 ± 00,017	00,190 ± 00,058
Complement C3	01,734 ± 00,392	00,238 ± 00,077	00,583 ± 00,393
Apolipoprotein H	00,153 ± 00,211	00,051 ± 00,053	00,630 ± 00,241
Vitronectin	16,399 ± 03,972	01,439 ± 00,200	01,551 ± 01,089
Haptoglobin	00,955 ± 00,373	00,172 ± 00,019	00,083 ± 00,055
Serotransferrin	00,122 ± 00,139	00,046 ± 00,051	03,643 ± 02,490
Thrombin	05,376 ± 01,309	00,736 ± 00,354	00,813 ± 00,606
Fetuin A	00,095 ± 00,024	00,021 ± 00,012	00,859 ± 00,259
Apolipoprotein B	02,721 ± 00,621	00,039 ± 00,021	00,220 ± 00,159
α-2-macroglobulin	00,936 ± 00,273	00,092 ± 00,065	00,016 ± 00,019
Protein AMBP	00,071 ± 00,018	00,011 ± 00,001	00,050 ± 00,025
Complement C1s	12,312 ± 05,916	01,748 ± 00,303	00,589 ± 00,426
Complement C1qB	28,943 ± 05,124	00,607 ± 00,124	00,062 ± 00,029
Angiotensinogen	00,087 ± 00,042	00,027 ± 00,009	00,041 ± 00,011
ITIH4	00,111 ± 00,063	00,030 ± 00,005	00,388 ± 00,258
Complement C1qA	36,711 ± 11,220	00,665 ± 00,092	00,057 ± 00,025
Complement C1qC	43,306 ± 08,055	00,742 ± 00,148	00,079 ± 00,014
Complement C1r	05,099 ± 02,051	00,488 ± 00,060	00,098 ± 00,040
Fibronectin	04,186 ± 02,865	00,324 ± 00,021	00,098 ± 00,027
Serum amyloid P-C	01,803 ± 01,033	00,455 ± 00,383	00,273 ± 00,265
Afamin	n.d.	00,001 ± 00,001	00,040 ± 00,015
Apolipoprotein(a)	00,079 ± 00,089	00,001 ± 00,001	00,075 ± 00,039
CD5 antigen-like	05,095 ± 01,459	00,123 ± 00,040	00,012 ± 00,005
Plasminogen	00,086 ± 00,071	00,012 ± 00,002	00,515 ± 00,241
Platelet basic protein	00,107 ± 00,077	00,004 ± 00,004	00,008 ± 00,011
Complement factor B	00,002 ± 00,001	00,002 ± 00,001	00,049 ± 00,022
C4b-binding protein α	00,507 ± 00,259	00,017 ± 00,003	00,006 ± 00,006
Complement C5	00,079 ± 00,016	00,003 ± 00,002	00,001 ± 00,001

AMBP, adipocyte plasma membrane-associated protein; IA, immunoaffinity; HGR, haptoglobin-related protein; IGHG1, immunoglobulin gamma-1 chain C region; IGκC, immunoglobulin kappa chain C region; ITIH4, Inter-alpha-trypsin inhibitor heavy chain H4; PLTP, Phospholipid transfer protein; PYCOX1, Prenylcysteine oxidase 1.

**Table 4:** Proteome of UC-HDL separated by size-exclusion chromatography

Protein Name	>12 nm	12–7.5 nm	<7.5 nm
	% of Total Protein (Mean ± Stdev)	% of Total Protein (Mean ± Stdev)	% of Total Protein (Mean ± Stdev)
Apolipoprotein A1	38,3334 ± 64,500	53,7352 ± 30,800	58,8046 ± 65,767
Apolipoprotein A2	24,1147 ± 73,584	28,1440 ± 15,436	79,256 ± 09,922
Apolipoprotein C1	36,929 ± 28,509	63,489 ± 07,788	05,860 ± 01,115
Apolipoprotein C2	26,747 ± 33,732	28,274 ± 06,390	07,655 ± 07,238
Apolipoprotein C3	13,093 ± 13,289	27,996 ± 09,241	30,796 ± 31,871
Apolipoprotein D	71,473 ± 35,833	26,704 ± 01,170	06,499 ± 03,265
Serum amyloid A4	08,685 ± 03,791	18,430 ± 01,952	03,737 ± 00,504
Paraoxonase 1	15,327 ± 02,404	07,212 ± 01,196	01,309 ± 00,485
Apolipoprotein M	02,384 ± 01,934	03,074 ± 00,340	00,281 ± 00,045
Apolipoprotein E	26,266 ± 17,062	02,245 ± 00,440	00,674 ± 00,534
Apolipoprotein F	00,273 ± 00,080	00,723 ± 00,113	00,261 ± 00,240
Albumin	33,372 ± 24,595	00,700 ± 00,596	23,7596 ± 46,127
IGκC	42,290 ± 17,297	00,421 ± 00,033	00,143 ± 00,018
Apolipoprotein A4	02,379 ± 02,672	00,340 ± 00,200	16,658 ± 01,531
Apolipoprotein C4	00,507 ± 00,512	00,336 ± 00,097	00,004 ± 00,004
Apolipoprotein L1	00,997 ± 00,940	00,268 ± 00,123	00,039 ± 00,029
IGHG1	09,090 ± 05,044	00,226 ± 00,067	00,273 ± 00,022
Transthyretin	00,644 ± 00,638	00,125 ± 00,062	00,541 ± 00,331
Clusterin	12,400 ± 06,306	00,124 ± 00,035	00,089 ± 00,065
α-1-antitrypsin	00,918 ± 00,860	00,100 ± 00,027	10,523 ± 04,252
PLTP	07,338 ± 02,583	00,092 ± 00,010	00,005 ± 00,003
LCAT	00,012 ± 00,010	00,053 ± 00,021	00,051 ± 00,037
PYCOX1	00,252 ± 00,220	00,041 ± 00,006	00,004 ± 00,005
HGR	00,659 ± 00,631	00,040 ± 00,013	00,002 ± 00,002
Complement C3	01,417 ± 00,517	00,037 ± 00,008	00,536 ± 00,607
Apolipoprotein H	00,534 ± 00,707	00,033 ± 00,028	01,085 ± 00,343
Vitronectin	28,742 ± 44,074	00,032 ± 00,022	00,210 ± 00,109
Haptoglobin	01,804 ± 01,643	00,027 ± 00,010	00,004 ± 00,002
Serotransferrin	00,910 ± 00,913	00,013 ± 00,017	03,651 ± 00,614
Thrombin	00,333 ± 00,286	00,010 ± 00,004	00,035 ± 00,036
Fetuin A	00,818 ± 00,712	00,009 ± 00,006	03,504 ± 01,975
Apolipoprotein B	02,761 ± 01,917	00,006 ± 00,003	00,027 ± 00,019
α-2-macroglobulin	08,869 ± 08,830	00,005 ± 00,007	00,006 ± 00,007
Protein AMBP	01,613 ± 01,174	00,005 ± 00,002	00,107 ± 00,066
Complement C1s	00,916 ± 00,608	00,005 ± 00,003	00,001 ± 00,001
Complement C1qB	01,245 ± 01,261	00,003 ± 00,004	00,002 ± 00,004
Angiotensinogen	00,108 ± 00,095	00,002 ± 00,001	00,251 ± 00,198
ITIH4	00,094 ± 00,096	00,002 ± 00,001	00,010 ± 00,008
Complement C1qA	01,156 ± 01,775	00,001 ± 00,002	00,001 ± 00,002
Complement C1qC	01,450 ± 02,111	00,001 ± 00,002	<00,001 ± 00,001
Complement C1r	00,231 ± 00,255	00,001 ± 00,001	<00,001 ± 00,001
Fibronectin	00,998 ± 01,367	00,001 ± 00,001	00,003 ± 00,005
Serum amyloid P-C	00,054 ± 00,093	00,001 ± 00,001	n.d.
Afamin	00,003 ± 00,004	00,001 ± 00,000	00,019 ± 00,001
Apolipoprotein(a)	00,382 ± 00,148	<00,001 ± 00,001	00,013 ± 00,001
CD5 antigen-like	01,777 ± 01,363	<00,001 ± 00,001	00,001 ± 00,002
Plasminogen	04,267 ± 06,898	<00,001 ± 00,001	00,084 ± 00,022
Platelet basic protein	01,741 ± 01,371	00,001 ± 00,000	00,076 ± 00,132
Complement factor B	00,014 ± 00,018	00,001 ± 00,000	00,072 ± 00,022
C4b-binding protein α	00,847 ± 01,365	<00,001 ± 00,000	<00,001 ± 00,000
Complement C5	00,402 ± 00,418	n.d.	<00,001 ± 00,000

AMBP, adipocyte plasma membrane-associated protein; HGR, haptoglobin-related protein; IGHG1, immunoglobulin gamma-1 chain C region; IGκC, immunoglobulin kappa chain C region; ITIH4, Inter-alpha-trypsin inhibitor heavy chain H4; PLTP, Phospholipid transfer protein; PYCOX1, Prenylcysteine oxidase 1; UC, ultracentrifugation.

**Table 5:** Proteome of DS-HDL separated by size-exclusion chromatography

Protein Name	>12 nm	12–7.5 nm	<7.5 nm
	% of Total Protein (Mean ± Stdev)	% of Total Protein (Mean ± Stdev)	% of Total Protein (Mean ± Stdev)
Apolipoprotein A1	36,8407 ± 44,815	64,5180 ± 19,456	47,7952 ± 13,4399
Apolipoprotein A2	18,1915 ± 49,076	21,2809 ± 31,816	18,741 ± 05,450
Apolipoprotein C1	37,787 ± 24,334	63,439 ± 15,598	02,116 ± 00,814
Apolipoprotein C2	14,327 ± 17,039	20,779 ± 04,686	04,392 ± 02,996
Apolipoprotein C3	06,476 ± 01,752	14,033 ± 07,623	14,621 ± 12,044
Apolipoprotein D	24,572 ± 16,866	04,078 ± 00,407	00,733 ± 00,216
Serum amyloid A4	12,968 ± 09,921	19,578 ± 02,707	01,055 ± 00,187
Paraoxonase 1	06,999 ± 01,023	02,732 ± 00,158	00,286 ± 00,120
Apolipoprotein M	01,562 ± 00,822	02,168 ± 00,045	00,079 ± 00,036
Apolipoprotein E	48,968 ± 30,634	02,555 ± 00,481	01,935 ± 00,959
Apolipoprotein F	00,126 ± 00,098	00,210 ± 00,011	00,227 ± 00,199
Albumin	07,952 ± 06,349	00,782 ± 00,659	15,5234 ± 52,565
IGκC	21,585 ± 16,432	00,637 ± 00,073	00,189 ± 00,016
Apolipoprotein A4	17,907 ± 02,534	06,583 ± 00,354	28,7272 ± 95,170
Apolipoprotein C4	00,478 ± 00,283	00,158 ± 00,061	n.d.
Apolipoprotein L1	01,343 ± 00,814	00,196 ± 00,098	00,025 ± 00,022
IGHG1	01,550 ± 01,267	00,424 ± 00,049	00,389 ± 00,133
Transthyretin	00,243 ± 00,241	00,061 ± 00,038	00,188 ± 00,122
Clusterin	10,318 ± 06,729	00,868 ± 00,156	02,296 ± 01,039
α-1-antitrypsin	00,450 ± 00,368	00,078 ± 00,045	03,073 ± 00,479
PLTP	02,622 ± 00,618	00,045 ± 00,003	00,002 ± 00,002
LCAT	00,008 ± 00,013	00,007 ± 00,000	00,046 ± 00,027
PYCOX1	00,107 ± 00,100	00,036 ± 00,007	00,011 ± 00,011
HGR	00,489 ± 00,434	00,024 ± 00,006	00,003 ± 00,002
Complement C3	04,385 ± 02,616	00,710 ± 00,175	01,123 ± 00,374
Apolipoprotein H	00,087 ± 00,126	00,052 ± 00,006	00,357 ± 00,146
Vitronecti	16,6531 ± 12,930	01,153 ± 00,520	01,451 ± 00,922
Haptoglobin	02,749 ± 02,390	00,041 ± 00,021	00,005 ± 00,001
Serotransferrin	00,100 ± 00,132	00,016 ± 00,018	03,864 ± 02,021
Thrombin	17,950 ± 16,047	00,188 ± 00,025	00,151 ± 00,007
Fetuin A	02,169 ± 02,349	00,114 ± 00,076	06,957 ± 03,425
Apolipoprotein B	00,202 ± 00,140	00,001 ± 00,000	00,010 ± 00,012
α-2-macroglobulin	11,761 ± 05,918	00,026 ± 00,018	00,010 ± 00,015
Protein AMBP	00,175 ± 00,180	00,027 ± 00,006	00,020 ± 00,009
Complement C1s	02,417 ± 01,183	00,016 ± 00,009	00,004 ± 00,002
Complement C1qB	00,167 ± 00,145	00,002 ± 00,003	00,006 ± 00,010
Angiotensinogen	00,032 ± 00,030	00,001 ± 00,001	00,034 ± 00,014
ITIH4	00,445 ± 00,424	00,021 ± 00,003	00,215 ± 00,029
Complement C1qA	00,242 ± 00,171	00,001 ± 00,001	00,006 ± 00,009
Complement C1qC	00,206 ± 00,257	00,001 ± 00,002	00,010 ± 00,017
Complement C1r	00,218 ± 00,179	00,005 ± 00,003	00,005 ± 00,006
Fibronectin	15,449 ± 12,742	00,079 ± 00,036	00,074 ± 00,091
Serum amyloid P-C	00,184 ± 00,163	00,004 ± 00,002	00,003 ± 00,006
Afamin	00,006 ± 00,009	00,002 ± 00,001	00,089 ± 00,039
Apolipoprotein(a)	00,003 ± 00,003	<00,001 ± 00,000	01,411 ± 00,756
CD5 antigen-like	02,889 ± 01,603	00,002 ± 00,001	00,021 ± 00,010
Plasminogen	00,495 ± 00,559	00,007 ± 00,002	10,985 ± 03,093
Platelet basic protein	00,348 ± 00,137	00,004 ± 00,004	01,872 ± 00,129
Complement factor B	00,005 ± 00,008	00,028 ± 00,007	00,388 ± 00,104
C4b-binding protein α	00,291 ± 00,423	00,002 ± 00,002	00,005 ± 00,003
Complement C5	01,334 ± 00,958	00,036 ± 00,015	00,059 ± 00,035

AMBP, adipocyte plasma membrane-associated protein; DS, dextran-sulfate precipitation; HGR, haptoglobin-related protein; IGHG1, immunoglobulin gamma-1 chain C region; IGκC, immunoglobulin kappa chain C region; ITIH4, Inter-alpha-trypsin inhibitor heavy chain H4; PLTP, Phospholipid transfer protein; PYCOX1, Prenylcysteine oxidase 1.

In comparison to total HDL, the proportion of apoA1 increased from 53.4%–59.8% to 53.7%–64.5% and for apoA2 increased from 18.0%–25.2% to 21.3%–28.1% in purified HDL_2/3_. The data from HDL_2/3_ suggests an increase in apoA2 and lower levels of apoA1 in UC-HDL (Table 5). Such an increase could be due to either a loss of apoA-I and thus an accumulation of apoA2, which is known to be less exchangeable than apoA1 (20) or from differences in the HDL subclass distribution across the isolation methods, since apoA2 is known to be preferentially associated with HDL_3_. The latter is certainly the case for DS-HDL, which has a much lower content of the smaller HDL_3_ subclass (Fig. 2). Looking at the apoA2/apoA1 ratio between the isolation methods, we found that the ratio was higher in UC-HDL compared to IA-HDL and DS-HDL (supplemental Table S3). Such an increase could be due to either a loss of apoA-I and thus an accumulation of apoA2, which is known to be less exchangeable than apoA1 (20) or from differences in the HDL subclass distribution across the isolation methods, since apoA2 is known to be preferentially associated with HDL_3_. The latter is certainly the case for DS-HDL, which has a much lower content of the smaller HDL_3_ subclass (Fig. 2). Notable differences between the isolation methods for HDL_2/3_ were a lower content of apoC’s in IA-HDL, while clusterin and PLTP were higher (Table 3). UC-HDL contained high levels of apoC3 (Table 4), while DS-HDL contained more apoA4 and low levels of apoD and paraoxonase when compared to the other methods (Table 5).

Notably, the content of a number of proteins decreased upon HDL_2/3_ purification in the range between 70% and 99%. A sharp decrease in protein abundance was observed for albumin (-86%), apoA4 (-73%), α-1-antytrypsin (-71%), apoB (-63%), serotransferrin (-87%), fetuin A (-90%), platelet basic protein (-99%), apo(a) (-88%), afamin (-65%), plasminogen (-90%), and a series of complement proteins (-60%-80%). After SEC separation, the majority of these proteins were either identified in the SEC fraction above 12 nm (most complement proteins, apo(a), apoB, platelet basic protein, plasminogen) or in the SEC fraction below 7.5 nm (α-1-antytrypsin, apoA4, fetuin A, afamin, serotranferrin) (Table 3, Table 4, Table 5). Our data suggest that the majority of these proteins is not present on the HDL_2/3_ subclass and more likely to be associated with other HDL subspecies or are co-isolates/contaminants of the isolation procedure itself.

**Fig. 3:**
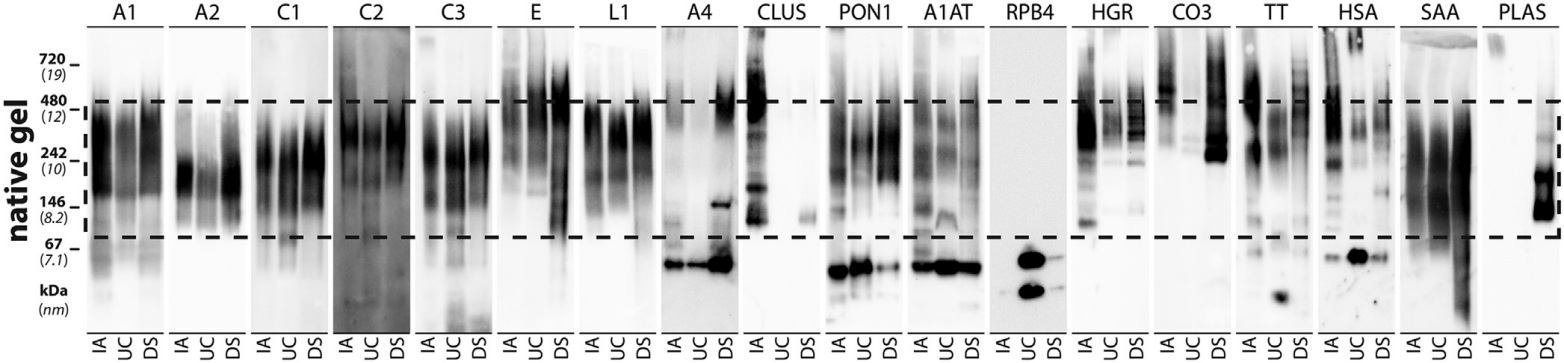
Detection of HDL-associated proteins after native-gel electrophoresis. Isolated HDL from immunoaffinity (IA), ultracentrifugation (UC), and dextran-sulfate (DS) precipitation isolation were loaded on 4%–16% native gels and separated by native gel electrophoresis. Gels were blotted and probed with specific antibodies: apoA1 (A1); apoA2 (A2); apoC1 (C1); apoC2 (C2); apoC3 (C3); apoE (E); apoL1 (L1); apoA4 (A4); clusterin (CLUS); paraoxonase 1 (PON1), α-1-antitrypsin (A1AT); retinol-binding protein 4 (RBP4); haptoglobin-related protein (HGR); complement C3 (CO3); transthyretin (TT); human serum albumin (HSA); serum amyloid A (SAA); plasminogen (PLAS). Dashed lines represent the usual size range of the main HDL_2/3_ subclass.

To further investigate the distribution of the identified proteins over the entire HDL size range, we performed native gel electrophoresis followed by Western blotting and antibody detection. The dashed line in Fig. 3 shows the size range of HDL_2/3_ between ∼7.5 and 12 nm. As expected, the highest abundance of apoA1 was observed throughout the HDL_2/3_ size range, while lower levels were visible at 5–7.5 nm indicating the presence of pre-β HDL in the isolates (Fig. 3). ApoA1 distribution was fairly even across HDL_2/3_ in IA-HDL and UC-HDL, while lower levels of apoA1 were observed in the smaller HDL_3_ fraction in DS HDL. This result is consistent with proteomics data (Table 2) and SEC analysis (Fig. 2). Other apoproteins such as apoA2, C1, C2, C3, E, and L1 showed a clear distribution consistent with the results of previous studies (21, 22). Analysis of the apoA4 distribution showed the presence of several different subgroups. One fraction had a size of around ∼7 nm and probably resembled the poorly-lipidated forms of apoA4 (21). This fraction was also removed by SEC and therefore explains the large decrease in apoA4 content after purification of HDL_2/3_ (Table 3). Two other apoA4 subgroups were present in the size range of 8 and 12 nm, respectively. Interestingly, these subgroups with sizes of 8 and 12 nm were not present in UC-HDL, suggesting that they either have different densities or are lost due to gravitational forces or osmotic pressure during UC. Clusterin was found mainly in IA-HDL with a distribution ranging from 7.5 up to 20 nm, suggesting that several subclasses of HDL carry clusterin. Paraoxonase 1 was present in HDL_2/3_ as well as in smaller particles around ∼6.5 nm. α-1-antitrypsin was present in HDL over the entire size range of HDL, with a significant accumulation at around 6.5 nm. Consistent with this observation, data from purification of total HDL into HDL_2/3_ showed a 70% reduction in α-1-antrypsin content (Table 1 vs. Table 2). This result suggests that 30% of the isolated α-1-antitrypsin was present in the size range of HDL_2/3_. Haptoglobin-related protein was detected in HDL from all three isolation methods in a size range of ∼10–13 nm. ApoA-I, apoL1, and haptoglobin-related protein together form the lipid-rich trypanosome lytic factor 1 (23), which is a part of the innate immune system responsible for the protection against African trypanosomes and Leishmania (22, 23). Western blot analyses suggest the presence of lytic trypanosome factor 1 in all three isolation methods. Complement C3 was present in IA and DS-HDL, while only traces were detectable in UC-HDL. Complement C3 has a molecular weight of ∼185 kDa, which corresponds to a large proportion of the complement C3 present in DS-HDL. Interestingly, IA-HDL and DS-HDL had a proportion of complement C3 in the size range ∼11–18 nm, well above the molecular weight of 185 kDa, suggesting interaction with HDL or other proteins. Transthyretin and albumin were detected in HDL by all three methods, especially in the size range of large HDL_2_. According to the proteomic data, albumin content was highest in UC-HDL, with the majority detected in the SEC traction below 7.5 nm smaller pre-β-HDL size fraction (Table 4). Serum amyloid A was present in all three isolated HDLs, covering the entire size range of HDL_2/3_. Of note, DS-HDL also contained serum amyloid A in the size fraction below 7.5 nm. Significant amounts of plasminogen were found only in DS-HDL in the size range of HDL_3_, while only small amounts were present in IA-HDL (Table 2). Western blot analysis from native gel electrophoresis can provide valuable insights into the distribution of proteins across different size ranges. However, due to the different nature of the isolation methods, direct comparison of the protein abundances between the different isolation methods was not possible. We found that IA purification of HDL resulted in more intense signals on Western blots, regardless of the actual protein load. Coomassie blue staining of native gels loaded with an equal amount of protein clearly showed that protein levels in the major HDL subclasses HDL_2/3_ were highest in UC-HDL (supplemental Fig. S1). In contrast, Western blot analysis of the content of apoA1 in these samples gave the highest signal in IA-HDL (Figs. 2 and S2). Elution of the bound protein in IA purification is performed with 3 M thiocyanate, a strong chaotropic agent, which disrupts antigen-antibody binding and releases the bound protein (24). Since thiocyanate is known to break hydrophobic, ionic, and hydrogen bonds, we hypothesize that the enhanced detection of proteins from IA-HDL is due to thiocyanate-induced accessibility of antibody binding sites.

### Impact of different isolation methods on the functionality of HDL

**Fig. 4:**
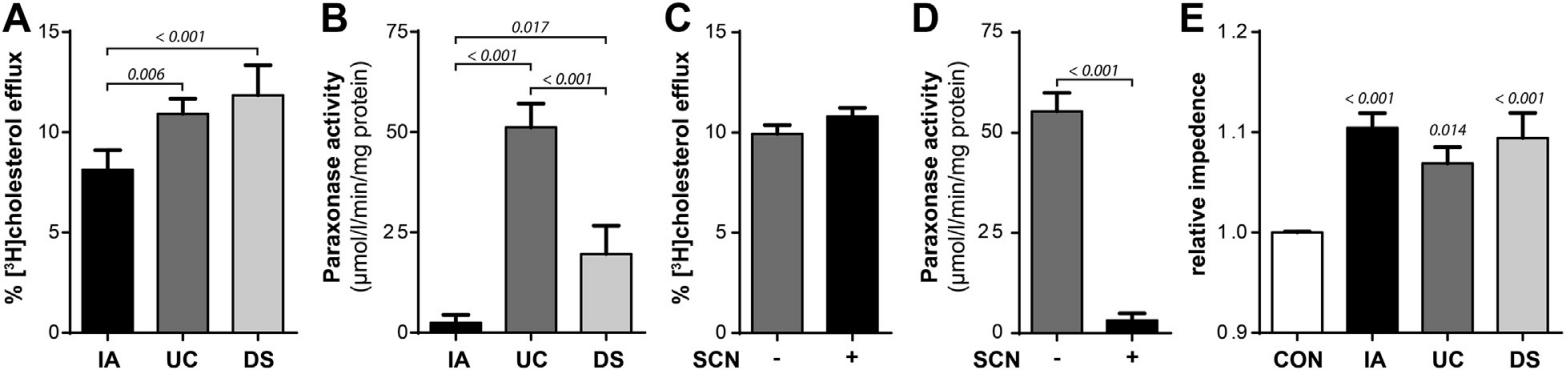
Functional characterization of HDL isolated by immunoaffinity (IA), ultracentrifugation (UC), or dextran-sulfate precipitation (DS). A: cholesterol efflux was assessed by incubating cAMP-stimulated J774.2 macrophages with 50 μg protein/ml HDL for 3 h. Cholesterol efflux is expressed as the radioactivity in the medium relative to total radioactivity in medium and cells. B: Arylesterase activity of HDL-associated paraoxonase was measured by using phenylacetate as substrate. C, D: UC-HDL was preincubated with 3M thiocyanate for 30 min. Subsequently, cholesterol efflux and paraoxonase activity were measured as indicated above. E: Endothelial barrier promoting activity of HDL was measured using an electrical impedance sensing system (ECIS). Ea.Hy926 cells were grown to a confluent monolayer and incubated with 100 μg/ml HDL and the impedance of the endothelial monolayer monitored over time. After 10 h impedance values were taken for quantification. All experiments were performed at least three times.

Next, we investigated whether the compositional differences observed in isolated HDL are linked to altered functionality. Cholesterol efflux was measured with the widely used system using cyclic adenosine monophosphate–treated J774 macrophages (25). Within this system cholesterol efflux is primarily mediated by apoA1 through ABCA1 (∼40%) and aqueous diffusion (∼50%) and to lower extant by SR-BI (∼10%) (25, 26). Importantly, we found that IA-HDL was significantly less effective at promoting cholesterol efflux from macrophages (Fig. 4A). Our data did not show a significant difference in apoA1 that would explain the reduction in cholesterol efflux from IA-HDL (Table 2). However, taking the SEC data showing the distribution of HDL subspecies into account (Fig. 2), we suggest that the reduced content of HDL_2/3_ might be involved in the reduction of cholesterol efflux capability. Interestingly, DS-HDL was most potent in promoting cholesterol efflux. Our data suggest an increased content of apoA1 together with an increased content of larger HDL_2_ particles as the prime causes. Paraoxonase 1 is an atheroprotective enzyme that is mainly bound to HDL in the circulation (27, 28, 29). We found that paraoxonase 1 activity was highest in UC-HDL, whereas activity was greatly reduced in DS-HDL and almost completely lost in IA-HDL (Fig. 4B). Importantly, the activity measurements were in striking contrast to the proteomics data, which indicated the highest level of paraoxonase 1 in IA-HDL (Table 2). We suspected that the elution conditions during IA purification may have caused the loss of function. To mimic the conditions during the elution process, we incubated UC-HDL with 3M thiocyanate and then removed it by gel filtration. This treatment resulted in an almost complete loss of paraoxonase-1 activity of UC-HDL (Fig. 4D). The reduced activity in DS-HDL was consistent with a lower paraoxonase-1 mass content (Table 2). We further tested whether thiocyanate also affected cholesterol efflux activity by treating UC-HDL with 3M thiocyanate. However, treatment of HDL with 3M-thiocyanate had no effect on cholesterol efflux capacity (Fig. 4C). We also analyzed the ability of HDL to promote endothelial integrity using an electrical impedance sensing system. We found that all three HDL isolates were able to improve endothelial barrier function by ∼10%. However, no significant differences were found between the three isolation methods, although UC-HDL tended to have a lower capacity (Fig. 3D).

## Discussion

While a variety of HDL isolation methods exist, their impact on the HDL proteome and its associated function remain largely unknown. In this study, we systematically compared the composition and function of HDL particles isolated by common methods such as IA, UC, and DS. Especially IA chromatography with apoA1-specific antibodies has emerged as an alternative method for isolating HDL because UC alters the composition of lipoproteins (30, 31). In contrast to UC and precipitation methods, IA purification isolates HDL across the full size spectrum of all apoA1-containing particles.

In the present study, we observed that IA-HDL contained subtypes that were completely absent in UC-HDL and DS-HDL and that differed in composition from the major forms of HDL. This is consistent with previous reports showing that apoA1 can form HDL subclasses with higher size ranges (32, 33). Further experiments will be necessary to distinguish whether these subclasses consist of one primary component or of a variety of different particles and how they function.

An interesting observation of the present study was that IA-HDL was less potent in promoting cholesterol efflux and almost completely lost its paraoxonase activity. Remarkably, the prime mediator of cholesterol efflux, apoA1, was more abundant in IA-HDL than in UC-HDL. Therefore, other factors like changes in the subclass distribution and in the lipidation status might be responsible for the impaired cholesterol efflux capacity. For ABCA1-mediated efflux, the size and lipidation status of the cholesterol acceptor is of critical importance, and small and lipid poor forms of HDL are more effective acceptors (34). We did not observe enrichment of small lipid-poor forms of HDL, such as pre-β HDL, in IA-HDL, so other factors seem more important. However, the differences in HDL subclass distribution were evident from SEC analysis. IA-HDL contained a fraction above 12 nm that made up about 20% of the overall protein mass. If this subclass were less efficient in promoting cholesterol efflux, an overall decrease would thus be plausible.

A further important observation of our study was that DS precipitation yielded HDL distinct of UC-HDL and IA-HDL. This is line with a recent report demonstrating that precipitations reagents can have a significant impact on the compositions and size of isolated HDL (35). DS does not to affect the size distribution of HDL but alters the quantity of a subset of apolipoproteins (35). However, DS seems to be less damaging to HDL than the widely used polyethylene glycol treatment of serum, which leads to significant changes in the size and in the apolipoprotein distribution of HDL (25).

SEC has been used as an alternative approach for the isolation of HDL (36, 37). While SEC is a method that allows purification of proteins in their native form, many plasma proteins have molecular weights in the same range as HDL, e.g., albumin dimer (∼135 kDa), IgG (150–180 kDa), and complement C3 (180 kDa) (13). In addition, many protein complexes overlap with the size distribution of HDL. Therefore, SEC alone is only able to enrich serum for HDL. To overcome this restrictions, SEC has to be combined with other methods to isolate HDL with high purity, for example with a lipid binding resin (38) or UC (39, 40, 41). Adding SEC after UC has the advantage that proteins that overlap with HDL in density, but are not HDL associated, are removed by size separation. The results of previous studies by others and us suggested that many of the detected proteins within UC-HDL isolates are not present in the HDL_2/3_ size range (39, 40). These proteins are found exclusively in the smaller pre-β sized fractions (39, 40) and cross-linking experiments revealed that no protein-proteins interaction with major HDL proteins are observed (39). When using a lipid binding resin to purify lipoproteins after SEC, it must be considered that other non-HDL plasma proteins with lipid-binding affinity are co-isolated. Moreover, re-solubilization of bound proteins requires enzymatic digestion. Therefore, the isolated lipoproteins cannot be used to study functional properties.

In conclusion, our data are the first to provide an in depth assessment of proteomic features of HDL isolated by UC, IA purification, and DS. We have demonstrated that the use of different isolation methods resulted in the isolation of HDL that was compositionally and functionally distinct. This is of particular importance as especially the use of IA purification gains widespread use for HDL isolation from clinical cohorts (42, 43, 44, 45). Special attention must be taken when IA isolated HDL will be used for functional assays as the elution conditions with 3M thiocyanate can significantly alter its functional properties. Our data showing that separation and purification of HDL subclasses have a profound effect on HDL structure and function may help in the selection of the most appropriate isolation method for experimental purposes.

## Data Availability

The data supporting this study are available in the article, the supplemental data, or available from the corresponding author upon reasonable request.

## Supplemental Data

This article contains supplemental data.

## Conflict of Interest

The authors declare that they have no conflict of interest.
