# Supplementary material for: HDL Isolated by Immunoaffinity, Ultracentrifugation, or Precipitation is Compositionally and Functionally Distinct
Source: J Lipid Res. 2022 Oct 29;63(12):100307. doi: 10.1016/j.jlr.2022.100307 (PMC9720336; doi:10.1016/j.jlr.2022.100307)
Supplement: Supporting information [file mmc1.docx]

Supplemental information

| **Supplemental table 1: List of antibodies** | | | | |
| --- | --- | --- | --- | --- |
| **Target protein** | **Supplier** | **Nr.:** | **Dilution** | **Host** |
| a-1-antitrypsin | Novus Bio | NBP1-78098 | 1:1000 | polyclonal rabbit |
| apoA-I | Academy Biomedical | 11A-G2b | 1:5000 | polyclonal goat |
| apoA-II | Academy Biomedical | 12A-G1b | 1:5000 | polyclonal goat |
| apoC-I | Academy Biomedical | 31A-G1b | 1:5000 | polyclonal goat |
| apoC-II | Academy Biomedical | 32A-G2b | 1:5000 | polyclonal goat |
| apoC-III | Academy Biomedical | 33A-G2b | 1:5000 | polyclonal goat |
| apoE | Academy Biomedical | 50A-G1b | 1:5000 | polyclonal goat |
| apoL-1 | Proteintech | 11486-2-AP | 1:1000 | polyclonal rabbit |
| apoA-IV | Proteintech | 17996-1-AP | 1:10000 | polyclonal rabbit |
| Clusterin | R&D Systems | MAB29372 | 1:1000 | monoclonal mouse |
| Complement C3 | ThermoFisher | PA1-29715 | 1:5000 | polyclonal goat |
| Haptoglobin | Eubio (LifeSpan) | LS-B13232 | 1:5000 | polyclonal goat |
| Paraoxonase-1 | Eubio (LifeSpan) | LS-C188035 | 1:5000 | polyclonal goat |
| Plasminogen | Academy Biomedical | PG60A-G1b | 1:5000 | polyclonal goat |
| Retinol-binding protein 4 | Abcam | ab13559 | 1:10000 | monoclonal rabbit |
| SAA | Courtesy of G. Kostner | RAS-H-SAA-A8 | 1:1000 | polyclonal goat |
| Transthyretin | Abcam | ab16006 | 1:1000 | polyclonal rabbit |
| Human serum albumin | Abcam | ab83465 | 1:1000 | polyclonal rabbit |

| **Supplemental table 2: Clinical characteristics of serum pools used for HDL isolation** | | | | | | |
| --- | --- | --- | --- | --- | --- | --- |
|  | n | m/f | age | Total cholesterol  (mg/dL) | HDL  (mg/dL) | LDL  (mg/dL) |
| Serum Pool-1 | 6 | 3/3 | 34.1 ± 8.8 | 201 ± 42 | 59 ± 6.2 | 128 ± 20 |
| Serum Pool-2 | 6 | 3/3 | 35.6 ± 6.0 | 214 ± 92 | 51 ± 7.3 | 146 ± 79 |
| Serum Pool-3 | 6 | 3/3 | 40.0 ± 9.6 | 202 ± 14 | 68 ± 7.1 | 119 ± 11 |

| **Supplemental table 3: Ratio of apoA2/apoA1 from DIA-MS/MS data** | | | | | | | | | |
| --- | --- | --- | --- | --- | --- | --- | --- | --- | --- |
|  | **IA** | | | **UC** | | | **DS** | | |
| Total HDL | 0.39 | ± | 0.04 | 0.47 | ± | 0.02 | 0.30 | ± | 0.02 |
| HDL_2/3_ | 0.43 | ± | 0.05 | 0.53 | ± | 0.05 | 0.33 | ± | 0.05 |


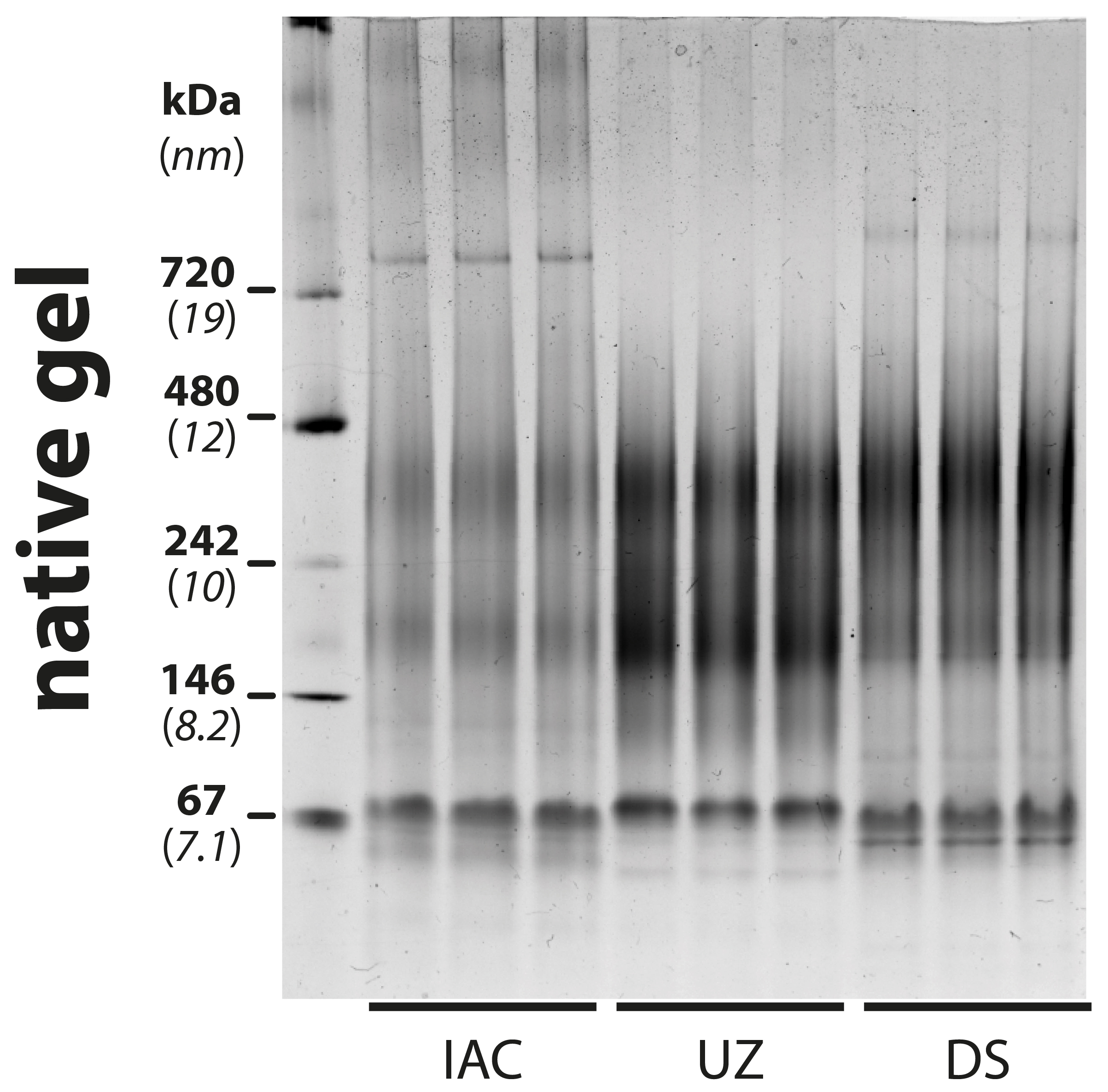


**Supplemental Figure 1: Immunoaffinity (IA), ultracentrifugation (UC) and dextran sulphate (DS) are reproducible methods for HDL isolation.** HDL was repeatedly isolated by the indicated methods from the same serum pool. Subsequently, HDL (5µg protein per lane) was separated by native gel electrophoresis on 4-16% gels. The gel was immediately stained with Commassie blue to visualize the protein load and size distribution.


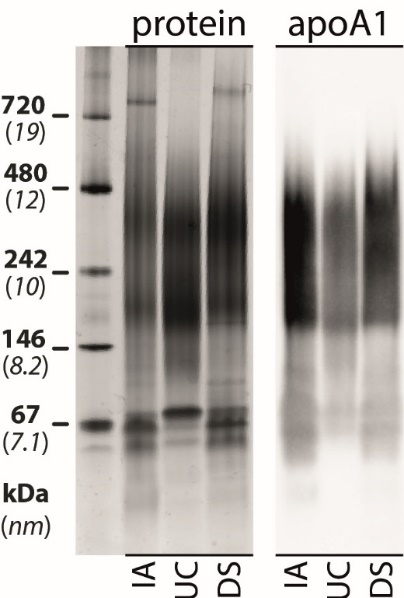


**Supplemental Figure 2: Comparison of HDL protein load between native gels and Western blots.** HDL (5µg protein per lane) was first separated by native gel electrophoresis on two 4-16% gels. The first gel was immediately stained with Commassie blue to visualize the protein load (left panel). The second gel was blotted onto a PVDF membrane and probed with a specific antibody against apoA1 (right panel). The right panel apoA1 was reused from Figure 3 in the main manuscript since experiments for protein loading and Western blotting were performed simultaneously. Immunoaffinity (IA), ultracentrifugation (UC), dextrane-sulphate (DS).
